# Altered neural connectivity during response inhibition in adolescents with attention-deficit/hyperactivity disorder and their unaffected siblings

**DOI:** 10.1016/j.nicl.2015.01.004

**Published:** 2015-01-13

**Authors:** Daan van Rooij, Catharina A. Hartman, Maarten Mennes, Jaap Oosterlaan, Barbara Franke, Nanda Rommelse, Dirk Heslenfeld, Stephen V. Faraone, Jan K. Buitelaar, Pieter J. Hoekstra

**Affiliations:** a Department of Psychiatry, University of Groningen, University Medical Center Groningen, Groningen, The Netherlands; b Centre for Cognitive Neuroimaging, Donders Institute for Brain Cognition and Behavior, Nijmegen, The Netherlands; c Department of Psychology, VU University Amsterdam, Amsterdam, The Netherlands; d Departments of Human Genetics and Psychiatry, Radboud University Medical Center, Nijmegen, The Netherlands; e Karakter Child and Adolescent Psychiatry, Radboud University Medical Center, Nijmegen, The Netherlands; f Departments of Psychiatry and of Neuroscience and Physiology, SUNY Upstate Medical University, Syracuse, NY, USA; g Department of Cognitive Neuroscience, Radboud University Medical Center, Donders Institute for Brain Cognition and Behavior, Nijmegen, The Netherlands

**Keywords:** ADHD, attention deficit/hyperactivity disorder, CD, conduct disorder, DMN, default mode network, GEE, generalized estimating equations, ICV, intraindividual coefficient of variance, ODD, oppositional defiant disorder, RD, reading disorder, ROI, region of interest, SSRT, stop-signal reaction time, SST, Stop-signal task, SI, supplementary information, WM, white matter, ADHD, PPI, Connectivity, Siblings, Response inhibition

## Abstract

**Introduction:**

Response inhibition is one of the executive functions impaired in attention-deficit/hyperactivity disorder (ADHD). Increasing evidence indicates that altered functional and structural neural connectivity are part of the neurobiological basis of ADHD. Here, we investigated if adolescents with ADHD show altered functional connectivity during response inhibition compared to their unaffected siblings and healthy controls.

**Methods:**

Response inhibition was assessed using the stop signal paradigm. Functional connectivity was assessed using psycho-physiological interaction analyses applied to BOLD time courses from seed regions within inferior- and superior frontal nodes of the response inhibition network. Resulting networks were compared between adolescents with ADHD (*N* = 185), their unaffected siblings (*N* = 111), and controls (*N* = 125).

**Results:**

Control subjects showed stronger functional connectivity than the other two groups within the response inhibition network, while subjects with ADHD showed relatively stronger connectivity between default mode network (DMN) nodes. Stronger connectivity within the response inhibition network was correlated with lower ADHD severity, while stronger connectivity with the DMN was correlated with increased ADHD severity. Siblings showed connectivity patterns similar to controls during successful inhibition and to ADHD subjects during failed inhibition. Additionally, siblings showed decreased connectivity with the primary motor areas as compared to both participants with ADHD and controls.

**Discussion:**

Subjects with ADHD fail to integrate activation within the response inhibition network and to inhibit connectivity with task-irrelevant regions. Unaffected siblings show similar alterations only during failed stop trials, as well as unique suppression of motor areas, suggesting compensatory strategies. These findings support the role of altered functional connectivity in understanding the neurobiology and familial transmission of ADHD.

## Highlights

• We investigate the neural connectivity during response inhibition using PPI.
• We investigate connectivity in participants with ADHD, their siblings and controls.
• Participants with ADHD show lower connectivity within the response inhibition network.
• Participants with ADHD show higher connectivity with the default mode network.
• Unaffected siblings show unique patterns of compensatory activation.

## 1 Introduction

Response inhibition, the process of actively suppressing an ongoing or inappropriate response, is considered one of the main cognitive control deficits underlying ADHD (Alderson et al., 2007; Goos et al., 2009; Crosbie et al., 2008, 2013). However, a recent meta-analysis has shown only moderate effect sizes and large heterogeneity in response inhibition performance in patients with ADHD, with half of the subjects showing no performance deficits (Lipszyc and R. Schachar, 2010). Brain activation during response inhibition, as measured by functional magnetic resonance imaging (fMRI), appears to be a more sensitive measure, as indicated by research in children (e.g. 12–14), adolescents (Katya Rubia et al., 2005), and adults with ADHD (Cubillo et al., 2011; Mulligan et al., 2011), including a study by our group (Van Rooij et al., 2014). These studies demonstrated that alterations within the neural networks responsible for cognitive control, inhibition, and attention can be found in the absence of behavioral response inhibition deficits. These alterations have been found even in unaffected siblings of subjects with ADHD (Van Rooij et al., 2014), adolescents with subthreshold ADHD (Whelan et al., 2012), and adults with ADHD (Cubillo et al., 2010).

Neuroimaging studies of response inhibition in healthy subjects have identified a highly interconnected neural network. This involves nodes from the frontal–striatal network such as the inferior frontal gyrus, pre-supplementary motor area, basal ganglia, and suprathalamic nucleus (A.R. Aron et al., 2007a,b; Zandbelt et al., 2013a,b; Hampshire et al., 2010; Majid et al., 2012; Sebastian et al., 2012; Swick et al., 2011; Verbruggen and G. Logan, 2008), as well as nodes from the frontal–parietal network including supramarginal and temporal/parietal areas (C. Fassbender et al., 2006; Chambers et al., 2009; Hugh Garavan et al., 2006; Simmonds et al., 2008). Functionally, the inferior frontal gyrus is involved in salience processing and initiation of the inhibition signal (A.R. Aron et al., 2007b; Cai et al., 2011; Chevrier et al., 2007; Hampshire et al., 2010; N. Swann et al., 2009a). This is thought to be the most likely site for integration of response inhibition and higher order cognitive control processes, executed from the superior frontal areas (A.R. Aron, 2011). The pre-supplementary motor area and subcortical regions on the other hand are thought to be involved in the execution of the stop processes (A.R. Aron et al., 2007a; Cai et al., 2012; Chao et al., 2009; de Wit et al., 2012; N.C. Swann et al., 2012; Tabu et al., 2011), whereas the parietal areas are thought to reflect attentional redirection and task-set maintenance during response inhibition (C. Fassbender et al., 2006; Chambers et al., 2009).

While each of these nodes plays a distinct role in response inhibition, the overall inhibition efficiency may depend on the degree of integration between the different parts of the network. Diminished functional connectivity between the left and right inferior frontal gyrus, caudate/thalamus, cingulate gyrus, and temporal/parietal regions during a response inhibition task has previously been found in adults with ADHD as compared to healthy controls (Cubillo et al., 2010). Additionally, evidence from structural (De La Fuente et al., 2013; N.C. Swann et al., 2012) and resting-state network studies (D.A. Fair et al., 2010; Mennes et al., 2011; Tian et al., 2006) have supported the necessity of network integration during response inhibition and have confirmed altered patterns of connectivity in subjects with ADHD. It is, therefore, specifically interesting to investigate to what extent the functional connectivity is altered in subjects with neural hypoactivation within the response inhibition network.

In a previous paper we showed decreased neural activation during response inhibition in left inferior frontal, left superior frontal, and bilateral temporal/parietal areas in adolescents with ADHD and their unaffected siblings as compared to healthy controls (Van Rooij et al., 2014). The primary aim of the current study was to investigate whether subjects with ADHD would also show decreased functional connectivity between these nodes of the response inhibition network and whether the degree of hypo-connectivity would be linked to ADHD severity. Secondarily, we aimed to investigate the familial nature of functional connectivity by comparing subjects with ADHD not only with healthy controls, but also with their unaffected siblings. Since unaffected siblings of subjects with ADHD share on average half of the genetic risk factors with their affected siblings, we expected similar but less extensive decreases in functional connectivity in this group (Bidwell et al., 2007; Crosbie et al., 2008, 2013). This would support the familial nature of decreased functional connectivity during response inhibition and its possible use as an endophenotype in ADHD. Finally, we aimed to investigate neural connectivity related to compensatory strategies in both subjects with ADHD and unaffected siblings. Previous investigations had suggested that subjects with ADHD may be able to recruit alternative neural recourses to compensate for deficits in prefrontal functioning (Catherine Fassbender and Schweitzer, 2006), although we previously did not encounter such compensatory mechanisms in our study sample with regard to neural activation (Van Rooij et al., 2014). We expected that compensation for deficits in neural connectivity within the response inhibition network might occur by recruiting compensatory resources in other brain regions, leading to increased connectivity with these areas.

## 2 Methods and materials

### 2.1 Participants

All subjects participated in the NeuroIMAGE project, the Dutch follow-up of the International Multicenter ADHD Genetics (IMAGE) study. Details about ethics approval, recruitment, assessment, and the general testing procedures can be found in the general methods and design paper of the NeuroIMAGE project (Von Rhein et al., 2014).

In short, ADHD diagnosis was based on semi-structured interviews (the Schedule for Affective Disorders and Schizophrenia for School-Age Children [K-SADS] (C. Kaufman et al., 1997)) as well as the Conners ADHD questionnaires (Conners et al., 1998a,b). Probands with ADHD had to have six or more hyperactive/impulsive and/or inattentive symptoms according to DSM-IV criteria (American Psychiatric Association, 2000); unaffected siblings and unrelated controls had to have less than two symptoms overall, based on a structured psychiatric interview (K-SADS) and Conners questionnaires.

Inclusion criteria for MRI participation consisted of the absence of claustrophobia and any metal in the body. Informed consent was acquired from all participants, with parents supplying consent for participants less than 16 years old. Subsequently, 208 participants with ADHD, 116 unaffected siblings, and 129 healthy controls successfully performed the stop signal task within an MRI scanner. Of these, 21 participants only completed three out of four response inhibition runs (12 subjects with ADHD and six unaffected siblings). Six participants were excluded after reaching an accuracy of <70% on the go-trials, indicating inadequate performance on the task and leaving an insufficient number of trials to estimate inhibition measures (four subjects with ADHD, two healthy controls). Eleven participants were removed after excessive movement (>3 mm within a single run) in the scanner (nine subjects with ADHD, one healthy control). Sixteen participants were excluded due to incidental neuroradiological findings. This led to a final inclusion of 185 subjects with ADHD, 111 unaffected siblings, and 124 controls in our analyses (see Table 1).

### 2.2 Stop signal task

A visual version of the stop signal task (Logan et al., 1984) was used to measure response inhibition during fMRI acquisition. In this task, participants had to respond as quickly as possible to a go-stimulus by left or right button press, unless shortly after presentation it was followed by a stop signal, in which case they were to withhold their response (25% of trials). The task difficulty was adaptive, meaning delays between the go- and stop stimulus were adjusted by 50 ms after every failed or successful response, leading to an approximately 50% success rate on the stop-trials for all subjects (except for the aforementioned six removed from the data). The task consisted of two practice blocks and four test blocks, each consisting of 60 trials.

The Stop Signal Reaction Time (SSRT) was the main measure of response inhibition efficiency, calculated by subtracting the eventual delay between the go and stop signals. Secondary task outcome measures were the intraindividual coefficient of variation (ICV; derived by dividing the reaction time variance by the mean reaction time), and the total number of errors. We included both omission and commission errors on go-trials in the error scores, since insufficient numbers of either event occurred to model them separately. Both secondary measures are related mainly to attentional processes that indirectly influence the response inhibition performance (Kofler et al., 2013; Schachar et al., 2004).

### 2.3 Task outcome analysis

To link functional connectivity to behavioral performance, the effects of diagnostic group (i.e. ADHD, vs. sibling, vs. healthy control) on the SST task-outcome measures were analyzed. This was analyzed using General Estimated Equations models in SPSS (SPSS 19.0 Inc.). Family affiliation was added as a between-subject factor to control for relatedness between participants. Age, gender, IQ, and scan-site were added as covariates. Effects of medication use and comorbid disorders such as oppositional defiant disorder, conduct disorder, and reading disorder on stop signal outcome measures were investigated in separate GEE models within the subjects with ADHD (see SI). Further details concerning the analysis of Stop Task outcomes can be found in Van Rooij et al. (2014).

### 2.4 fMRI acquisition

Data were acquired at two scanning locations on similar 1.5 Tesla Siemens scanners (Siemens Sonata at VU UMC in Amsterdam; Siemens Avanto at Donders Center for Cognitive Neuroimaging in Nijmegen) using identical protocols, using a T2*weighted echo planar imaging sequence (TR = 2340 ms, TE = 40 ms, FOV = 224 × 224 mm, 37 interleaved slices, voxel size = 3.5 × 3.5 × 3.5 mm, 94 volumes per run). Each participant's MPRAGE T1 scan (TR = 2730 ms, TE = 2.95 ms, TI = 1000 ms, voxel size = 1 × 1 × 1 mm, FOV = 256 mm, 176 slices) was used for spatial localization and normalization.

### 2.5 Selection of regions of interest

**Fig. 1:**
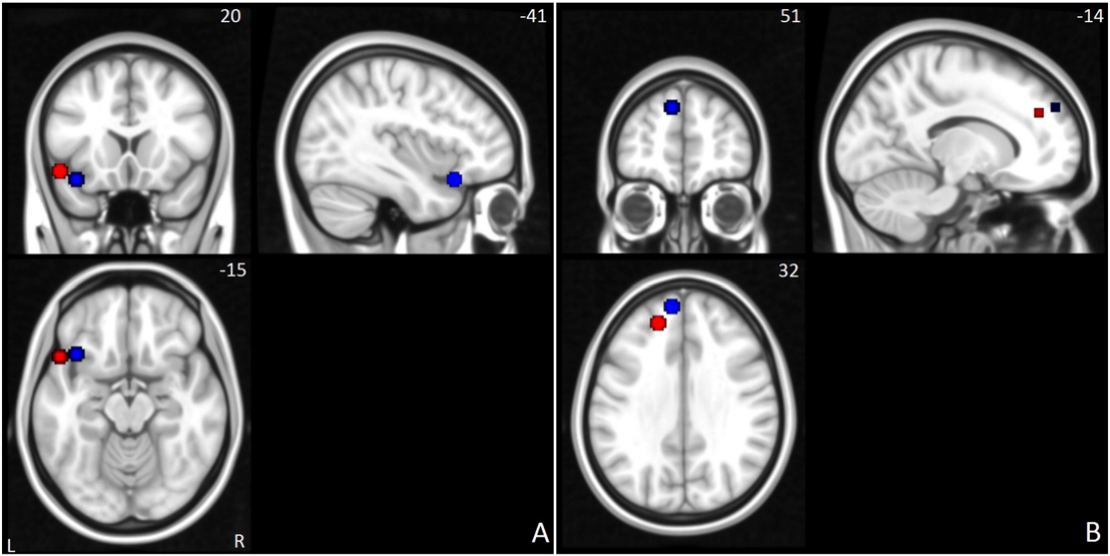
Region of interest (ROIs) based on the maximal diagnostic group difference in neural activation (ADHD vs. Siblings vs. Controls). ROIs of the inferior frontal gyrus (A) and superior frontal gyrus (B). Red spheres indicate the seed regions from the failed-stop contrast, blue spheres indicate ROIs from the successful-stop contrast.

To investigate functional connectivity, several regions of interest (ROIs) were defined as seed regions. Instead of basing our selection of ROIs on meta-analysis and data from healthy control studies (Hart et al., 2013), we selected ROIs based on the brain regions showing peak neural activation differences between probands with ADHD and controls in a previous study from the same sample (Van Rooij et al., 2014). This is because we aimed at further investigating possible altered connectivity between these diagnostic groups, extending and complementing thereby our previous analysis on neural activation differences. Details regarding the procedure and analyses of the fMRI analysis of the stop-task detailing the differences in neural activation between probands with ADHD and healthy control can be found in the original publication, currently under revision (Van Rooij et al., 2014). In short, two conditions of interest where defined, failed stop–go and successful stop–go trials. These conditions reflect the neural correlates of both failed and successful inhibitions, using the go-trials as an implicit baseline. In both conditions, similar patterns of between group activation differences were found, with the strongest activation differences located in the left inferior and left superior frontal gyri. These results can be found in the supplementary information. Previous literature showed the inferior frontal gyrus to be crucial for the initiation of the stopping process, while superior frontal gyrus is associated with top-down control over response inhibition (A.R. Aron, 2011; Chambers et al., 2009; Hugh Garavan et al., 2006; N.C. Swann et al., 2012; Simmonds et al., 2008). Therefore, to investigate possible functional connectivity differences between diagnostic groups during response inhibition, a total of four ROIs were defined based on the voxels with peak activation differences in left inferior frontal gyrus and superior frontal gyrus, from both the successful and failed stop conditions (see Table 2, Fig. 1).

### 2.6 Psycho-physiological interaction connectivity analysis

A psycho-physiological interaction analysis executed in FSL FEAT (FMRIB's Software Library, http://www.fmrib.ox.ac.uk/fsl; fMRI Expert Analysis Tool, version 6.0) was used to determine which voxels co-varied in activation with the seed ROI as a function of task condition, with the valence of the covariance coefficient indicating positive or negative connectivity. The average time series of neural activation was extracted from 6 mm diameter spheres around each ROI and entered as a physiological variable in the psycho-physiological interaction model. The task contrast of interest (successful stop–go or failed stop–go trials) was entered as a psychological variable. The psycho-physiological interaction was obtained by modeling a third variable as the interaction term between the latter two variables. Since the main task-contrast is included in the design matrix, the connectivity is effectively calculated over the residuals of the activation maps, ensuring orthogonality of the connectivity data from activation data. For optimal estimation of movement artifacts, the 24 realignment parameters from the first-level analysis were added, as well as spike regressors for all events within 8 s preceding peak movements greater than 1 mm. Runs with total movement exceeding 3 mm were removed from the analysis. To correct for background noise, the signal from cerebral spinal fluid (CSF) and white matter (WM), extracted using FSL CSF and WM probability masks (threshold of >0.8) were also added in the first level design. Age, gender, IQ, and scan-site were included as covariates.

An F-contrast comparing the control group with the other two groups was applied to the psycho-physiological interaction variable, providing *z*-maps detailing the between-group effect on functional connectivity. Multiple comparisons of resulting *z*-maps were performed by FSL standards using thresholding clusters with a minimum *z*-score of 2.3 and a corrected *p*-value of <0.05 (Woo et al., 2014). Between-group differences were further investigated by exporting the average connectivity values of all clusters that reached significance in the *F*-tests, and analyzing these in separate models in SPSS to account for the familial relations between siblings within our sample. These post-hoc analyses were used to determine the size and direction of any differences in functional connectivity between subjects with ADHD, their unaffected siblings, and healthy controls. Bonferroni–Holm corrections were implemented to account for multiple testing in all post-hoc tests (Holm, 1979).

A series of sensitivity analyses were run, given that the participants with ADHD, unaffected siblings, and controls in our study were not a-priori matched on demographic factors and across scanner sites (see also Von Rhein et al., 2014). Therefore, the potential confounding effects of IQ, gender, scanner location, and age were analyzed to validate the robustness of the main diagnostic group effects. These analyses, together with tests for the influence of comorbid disorders and medication use in subjects with ADHD are also described in the Supplementary Information (SI). To ensure potential motion effects did not influence the group comparison, we calculated the root-mean-square of the frame-wise displacement over all runs per subject; the three diagnostic groups did not differ significantly on this measure (χ^2^ = 4.46; *p* = .107). The association between frame-wise displacement and the connectivity values from the nodes indicated in the group contrasts is depicted in supplementary Table 5.

Finally, we investigated if functional connectivity was associated with response inhibition performance or with ADHD severity. Two sets of GEE analyses were performed; one to test the association between the SST outcome measures and connectivity in the significant nodes from the group contrast and a second to test the associations between ADHD severity, as measured by the *T*-score of the Conners questionnaire, and these connectivity patterns. Age, gender, IQ, and scan-site were also added as covariates in the post-hoc analyses.

## 3 Results

### 3.1 Task outcome measures

Significant effects of diagnostic group were found on all SST outcome measures (see Table 1). SSRT was slower in subjects with ADHD (mean = 269 ms) as compared to both unaffected siblings (mean = 254 ms, *p* = .015) and healthy controls (mean = 255 ms, *p* = .05), but did not differ between the latter two groups. ICVs were higher in subjects with ADHD (mean = 0.2082) than in unaffected siblings (mean = 0.1860, *p* < .001), who showed more variability than controls (mean = 0.1743, *p* < .031). Subjects with ADHD made more errors (mean = 6.4) than siblings (mean = 4.2, *p* < .013), who made more errors than controls (mean = 3.1, *p* < .032). No effects of gender, and IQ were found on any of the SST measures, nor did comorbid diagnoses or medication status affect results. Age had a significant main effect on SST performance, though no interaction effects of age with diagnostic group were found (see SI).

### 3.2 Task connectivity patterns

**Fig. 2:**
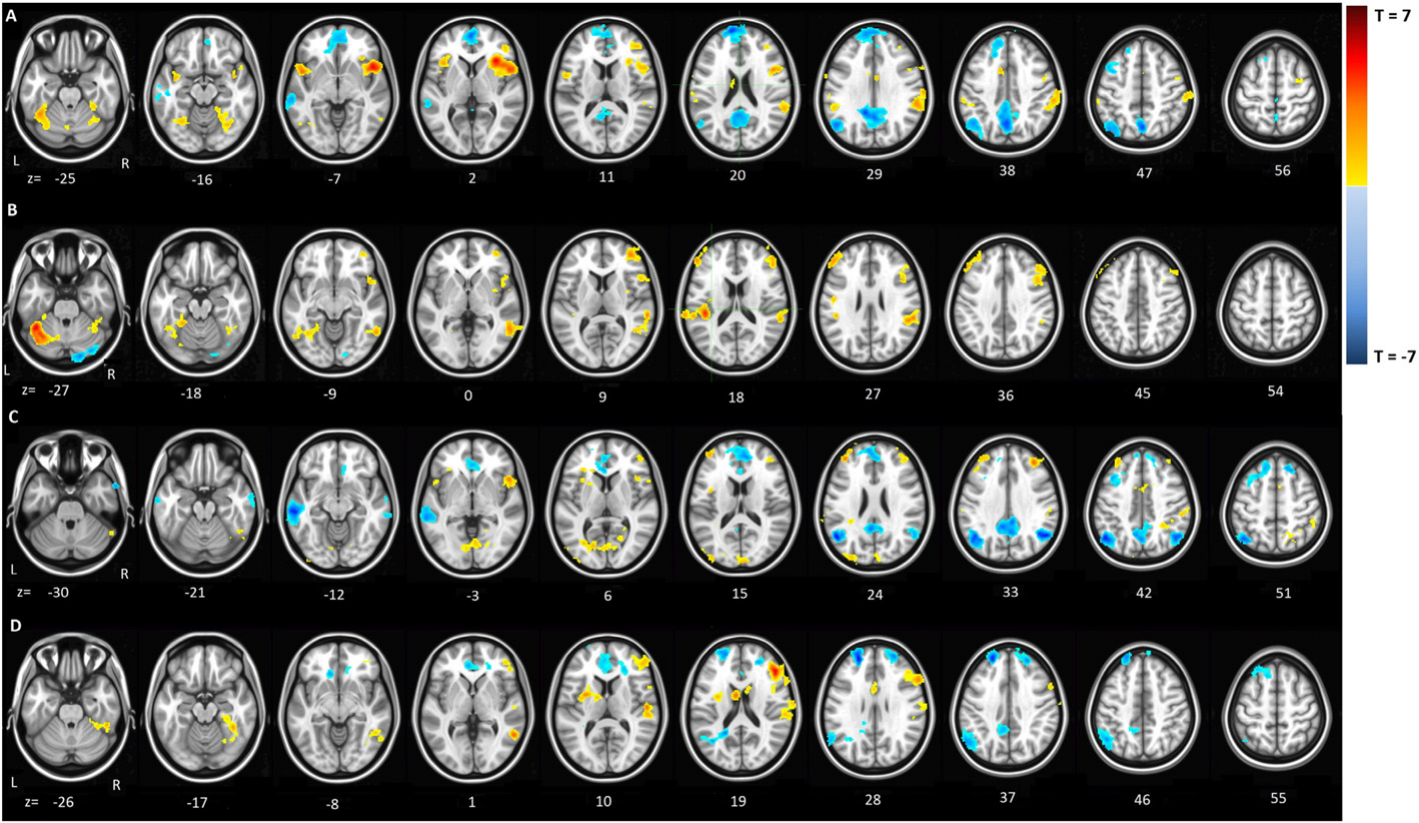
Functional connectivity patterns from the inferior frontal seed node, during successful stop condition (A) and failed stop condition (B); and functional connectivity from the superior frontal seed node, during successful stop condition (C) and failed stop condition (D). Red/yellow hues indicate positive connectivity values; blue hues indicate negative connectivity values.

Average connectivity patterns over all subjects from the left inferior frontal seed region for the successful stop network and failed stop conditions are shown in Table 3 and Fig. 2A and B. The connectivity patterns from the superior frontal seed are shown in Table 4, and Fig. 2C and D. Over all subjects and task conditions, the areas that show positive connectivity with the seed regions during stop-task performance mainly encompass the inferior frontal, anterior cingulate, basal ganglia and supramarginal nodes. Negative connectivity with the seed regions is found in precuneus, occipital and medial frontal areas.

### 3.3 Group differences in connectivity patterns

**Fig. 3:**
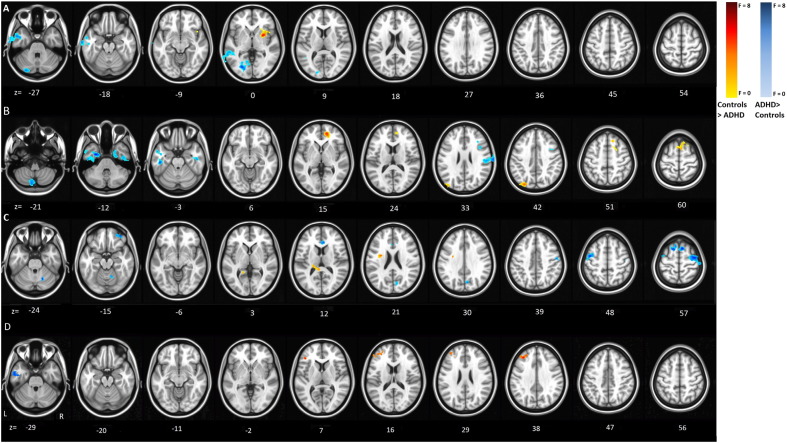
Differences in functional connectivity based on *F*-test comparing all three diagnostic groups. Connectivity patterns depicted from the inferior frontal seed region, in the successful stop condition (A) and the failed stop condition (B); as well as from the superior frontal seed region, in the successful stop condition (C) and the failed stop condition (D). Red hues indicate significantly higher connectivity in controls; blue hues indicate higher connectivity in ADHD subjects or unaffected siblings.

The group differences (i.e. controls vs. probands with ADHD vs. siblings) in connectivity patterns from the left inferior frontal seed regions are depicted in Table 5 and Fig. 3A and B. Additional visual representation of the group differences within each node can also be found in the SI. In Fig. 3, for illustration purposes, nodes with higher connectivity values in controls are depicted in red-yellow and nodes with higher connectivity values in probands or siblings in blue-white. These results indicate that control subjects showed increased functional connectivity with the right basal ganglia during successful stop trials, as well as increased connectivity between the left and right inferior frontal gyrus, superior frontal gyrus, and pre-supplementary motor area during the failed stop condition, as compared to both other groups. Subjects with ADHD had stronger connectivity between the left inferior frontal seed and bilateral temporal poles and cerebellum in both conditions and with the right supramarginal gyrus during failed-stop trials as compared to controls. Unaffected siblings showed similar connectivity patterns from the left inferior frontal seed as controls during the successful stop condition and similar connectivity as subjects with ADHD during the failed stop condition. Additionally, during the failed stop condition the unaffected siblings showed unique hypo-connectivity with the medial frontal gyrus as compared to subjects with ADHD and healthy controls.

The group differences in the connectivity between healthy controls and ADHD probands or siblings from the superior frontal seed region are shown in Table 6 and Fig. 3C and D. These results indicate that controls had stronger connectivity with the thalamus and operculum during the successful stop condition and with the left inferior frontal gyrus in the failed stop condition as compared to both other groups. Subjects with ADHD showed stronger connectivity of the superior frontal seed with medial frontal, precuneus during successful stops and with temporal areas during failed stops as compared to controls. Unaffected siblings again showed similar connectivity patterns as controls from the superior frontal seed region during the successful stop condition, together with unique hypo-connectivity with the precentral and primary motor areas as compared to both other groups. During the failed stop condition, they showed similar hypo-connectivity as subjects with ADHD with the middle frontal gyrus and similar connectivity as controls with the left inferior frontal gyrus.

The Cohen's d values from Tables 5 and 6 range from 0.315 to 0.628, with an average of 0.425, indicating moderate effect sizes for the diagnostic group effects, though there is still considerable overlap in the observed PPI connectivity values between the three diagnostic groups.

No main or interaction effects with group of the covariates IQ, gender, and scan-site were detected within these between-group analyses. Several main effects of age were found, but no significant interaction effects of age with diagnostic group either. Nevertheless, in the SI, findings from several additional sensitivity analyses were added to document the potential influence of these covariates, as well as of medication duration and comorbid disorders. These sensitivity analyses indicated our main effects did not change when these factors were incorporated in the analyses. Connectivity between the left inferior frontal seed and posterior middle temporal as well as middle frontal areas was associated with the average frame-wise displacement values, although these associations did not survive multiple comparisons. Nevertheless, the group comparisons in these nodes was adapted to include the frame-wise displacement as an additional factor in the model, to ensure the between-group results were controlled for motion effects (see Table 5).

### 3.4 Association between connectivity patterns and ADHD severity scores

Connectivity strength between the left inferior frontal seed region and all other regions was significantly associated with ADHD severity except for connectivity with the middle temporal, occipital, and medial frontal gyrus. Inspection of the B-values from these tests indicated that connectivity strength between the inferior frontal gyrus seed and pre-supplementary motor area was negatively correlated with ADHD severity, while connectivity with the temporal pole, precuneus, and cerebellum was positively correlated with ADHD severity.

Connectivity of the left superior frontal seed regions with all other regions except the inferior frontal node was also significantly associated with ADHD severity. B-values indicated negative correlations between thalamus and operculum and ADHD severity, while the nodes in temporal, cerebellum, and precuneus areas were positively correlated with ADHD severity (see SI Table 4).

### 3.5 Association between connectivity patterns and stop-task outcome measures

Several associations were found between connectivity measures in the nodes indicated in the group-contrast and stop-task outcome measures. Specifically, connectivity between the left inferior frontal seed and the anterior middle temporal gyrus was positively associated with ICV and SSRT (B = .792, *p* < .001, *R*^2^ = .044; B = .009, *p* = .009, *R*^2^ = .011; respectively) in the successful-stop condition. Thus, increased connectivity was related to higher variability and poorer response inhibition performance. In the failed-stop condition, a positive association between inferior frontal and medial frontal connectivity and error rates was found (B = .003, *p* = .019, *R*^2^ = .006), indicating that increased connectivity between these regions was associated with worse task performance, though this latter result did not survive the Bonferonni–Holm correction for multiple-comparisons.

Connectivity between the superior frontal seed region and thalamic connectivity was negatively associated with error rates (B = .002, *p* = .005, *R*^2^ = .05). Operculum connectivity was additionally negatively correlated with SSRT (B = −.022, *p* = .031) during successful stop trials, though this result did not survive Bonferonni-Holm correction. In other words, higher thalamus connectivity was associated with better task performance (see SI Table 3).

## 4 Discussion

Using psycho-physiological interaction analysis to investigate functional neural connectivity patterns during response inhibition, the current study provided evidence for altered functional connectivity patterns underlying response inhibition in adolescents with ADHD and their unaffected siblings, compared to healthy controls. Behavioral response inhibition deficits were only present in subjects with ADHD, as reported previously (Van Rooij et al., 2014).

Task related connectivity over all subjects in the successful-stop condition showed positive connectivity between the left inferior frontal and superior frontal seed regions with the right inferior frontal gyrus, basal ganglia, thalamus, and supramarginal areas, indicating strong connectivity within the response inhibition network and nodes belonging to the ventral attention network (Cortese et al., 2012). Negative connectivity was observed between seed regions and nodes in the medial frontal, precuneus, and temporal areas, which are generally attributed to the default mode network (DMN). During the failed-stop condition, positive connectivity patterns remained relatively stable, while negative connectivity patterns were largely reduced. These results provide evidence that the integration of the response inhibition and attention networks is key for proper response inhibition and support previous findings on the role of these networks in response inhibition (B.B. Zandbelt et al., 2013a,b; Chevrier et al., 2007; D.J. Sharp et al., 2010; Jahfari et al., 2011; M.C. Stevens et al., 2007; N.C. Swann et al., 2012). Additionally, recent studies have shown that suppression of activation in irrelevant networks, such as the DMN, is necessary for successful task performance (Fox et al., 2005; Gao and Lin, 2012; Spreng et al., 2010). The pattern of negative correlations between seed regions and task-irrelevant nodes during successful versus failed inhibitions in our study suggests that suppression of irrelevant networks is key for proper response inhibition.

When compared with controls, subjects with ADHD showed weaker connectivity within the response inhibition network and stronger connectivity between the seed regions and nodes in temporal cortex and precuneus. This pattern of increased and decreased connectivity in adolescents with ADHD largely matches the pattern of positive and negative task related connectivity described above, i.e. subjects with ADHD showed weaker integration between the relevant nodes in the response inhibition network than controls and stronger connectivity with DMN nodes, which are irrelevant for task performance. The continued functional connectivity with task irrelevant nodes is a likely source of interference and may cause poorer task performance in these subjects (Hampson et al., 2010), as has previously been indicated in several other disorders (H. Liu et al., 2012; Hamilton et al., 2011). This interpretation is also supported by the associations between connectivity and ADHD severity. The direction of these associations followed the same direction as the group contrasts, with higher frontal, opercular, and subcortical connectivity related to lower ADHD severity and higher posterior connectivity related to higher ADHD severity. This indicates, in line with our hypothesis, that increased connectivity with DMN nodes was related to higher ADHD severity, while connectivity with nodes within the functional response inhibition network was related to lower severity. The exception within this pattern of results was the stronger connectivity with cerebellum shown by subjects with ADHD, which was also related to more severe ADHD symptoms. However, previous studies in healthy subjects have indicated a role for the cerebellum in the frontal-striatal-cerebellar network during response inhibition (H. Garavan et al., 2003, 1999; Mostofsky et al., 2003), while other studies have indicated decreased cerebellar volumes in children with ADHD (Ivanov et al., 2014; Mackie et al., 2007). More research will be required to specifically delineate whether this additional connectivity with the cerebellum in probands reflects compensatory strategy during response inhibition, or is unrelated to response inhibition performance and associated with decreased cerebellar volumes.

Our analyses of the relationship between behavioral task outcome measures and connectivity further supports the potential functional importance of proper integration and suppression, as connectivity with the thalamus and operculum was related with better task performance, and medial temporal activation with worse performance. Medial frontal activation was also related with worse performance, although this may be related to increased error monitoring activation after failed inhibition (Van Meel et al., 2007). However, effect sizes of these relations were small, and connectivity from other nodes did not significantly correlate with performance. Further research should establish which factors determine this potential relation between connectivity and task performance.

In unaffected siblings, the observed pattern of connectivity was almost identical to the healthy controls in the successful-stop condition, while during the failed-stop condition the patterns resembled those of subjects with ADHD. This pattern of partially overlapping hypo-connectivity between subjects with ADHD and their siblings supports the familial nature of functional connectivity, and is in line with our hypothesis regarding shared genetic risk factors between subjects with ADHD and their siblings and supports the utility of neural measures of response inhibition as a putative endophenotype for ADHD. Moreover, siblings showed partly unique patterns of functional connectivity between the seed regions, medial frontal, and motor areas as compared to both other groups. Since these unique patterns of hypo-connectivity are all located in task-irrelevant nodes, and since the connectivity values in these nodes are all positively associated with ADHD severity, we argue that the increased suppression of these areas may constitute a compensatory mechanism for decreased integration of the response-inhibition. Specifically, the primary motor areas are a main downstream target of the response inhibition network (Aron and Poldrack, 2006; Aron et al., 2007b; Aron, 2011), suppression of which is necessary for motor inhibition (Swann et al., 2009b; Stinear et al., 2009). Stronger inhibition of the primary motor areas may provide unaffected siblings with an alternative strategy to achieve appropriate levels of inhibition, distinct from the response inhibition network proper. In our previous study, no compensatory neural activation during response inhibition was found in unaffected siblings. The current results therefore suggest that compensatory connectivity may be able to offset hypoactivation in the response inhibition network.

The hyper-connectivity shown by subjects with ADHD and siblings between the left inferior frontal seed and right supramarginal gyrus also warrants further attention. The supramarginal areas are considered part of the ventral attention network (Cortese et al., 2012; Dosenbach et al., 2008), and show generally positive connectivity with the response inhibition network over both conditions. Previous studies have attributed increased neural activation in supramarginal areas during response inhibition to compensatory activation utilized by subjects with ADHD to normalize task performance (Dillo et al., 2010; Durston et al., 2003; Karch et al., 2010). However, this explanation cannot directly be extrapolated to the current data, as we found no relation between connectivity with supramarginal areas and task outcome measures and only observed increased connectivity in subjects with ADHD during failed but not successful stop trials. It is therefore unclear from the current data if enhanced connectivity with the supramarginal areas in participants with ADHD and their siblings reflects the recruitment of additional neural resources, beneficial to the response inhibition process, or an additional source of unrelated or interfering activity.

Several interesting observations can be made when comparing the current group differences in PPI connectivity with our previously reported activation differences in the same sample (Van Rooij et al., 2014). The connectivity and activation data have similar effect sizes (the average Cohen's d for connectivity betas is 0.425 and the average Cohen's d for clusters reported in the previous activation research was 0.407). Since the PPI analysis is corrected for the main task-contrast, the resulting correlation between PPI beta values from any nodes with any beta values from the task activation was as low as −0.02 (SD = 0.04). This indicates that both the connectivity and activation parameters uniquely explain variance in ADHD severity. These observations, taken together with abovementioned unique patterns of negative connectivity as well as compensatory connectivity patterns in unaffected siblings both unseen in the activation data, further support the added value of employing both activation and connectivity analyses within fMRI research.

Our study and its findings should be viewed in the context of its strengths and weaknesses. Clear strengths of the current paper are the large sample size, as well as the inclusion of unaffected siblings in the design, which provides insight into the familial nature of functional connectivity patterns. However, our current analyses do not allow inferences about causal pathways within the response inhibition network and the specific role of the ventral attention network in response inhibition. Future studies might use causal connectivity models (Stevens et al., 2007) or interfering transcranial magnetic stimulation (Zandbelt et al., 2013a,b) in connected nodes to dissociated these pathways.

In conclusion, we showed hypo-connectivity during response inhibition in both adolescents with ADHD and their unaffected siblings along with concomitant hyper-connectivity with DMN nodes in adolescents with ADHD with possible compensatory mechanisms in their unaffected siblings. Additionally, we showed that the degree of functional connectivity in the response inhibition network is correlated with ADHD symptom severity. We conclude that altered functional connectivity may represent a significant part of the neurobiological alterations underlying ADHD.

## Financial disclosures

This work was supported by NIH Grant R01MH62873 (to Stephen V. Faraone), NWO Large Investment Grant 1750102007010 (to Jan Buitelaar), and grants from Radboud University Nijmegen Medical Center, University Medical Center Groningen and Accare, and VU University Amsterdam. Jan K. Buitelaar has been in the past 3 years a consultant to/member of advisory board of/and/or speaker for Janssen Cilag BV, Eli Lilly, Bristol-Myer Squibb, Schering-Plough, UCB, Shire, Novartis, and Servier. He is not an employee of any of these companies and not a stock shareholder of any of these companies. He has no other financial or material support, including expert testimony, patents and royalties. Jaap Oosterlaan has received in the past 3 years an investigator initiated grant from Shire pharmaceuticals. Pieter Hoekstra has been paid consultant of Shire and Eli Lilly and has received unrestricted research funding from Shire.

## Acknowledgments

We acknowledge the Department of Pediatrics of the VU University Medical Center for having the opportunity to use the mock scanner for preparation of our participants. The authors thank Roshan Cools for her invaluable input and comments in the preparation of this manuscript.

## Figures and Tables

**Table 1:** Participant characteristics and task outcomes derived from the SST.

	ADHD		Siblings		Controls		Wald-χ^2^	Cohen's *d*	*p*-Value	Between group effects
Males	129		48		55					
Females	56		63		69		**28.1**	**.536**	**<.001**	ADHD < Sibs = Controls
	Mean	SD	Mean	SD	Mean	SD				
ADHD symptoms a	12.9	3.1	1.3	3.4	0.6	1.5	**242.7**	**2.34**	**<.001**	ADHD > Sibs = Controls
Age (year)	17.3	3.2	17.3	4	16.5	3.3	1.6	.124	.44	
Estimated IQ b	95.3	16.8	102.4	15.9	107.1	14.5	**38.2**	**.633**	**<.001**	ADHD < Sibs < Controls
Education (yr)	12.82	2.14	12.82	2.22	13.52	1.91	**6.387**	**.249**	**.041**	ADHD = Sibs < Controls
Age range	8–25		8–27		9–23					
IQ range b	55–138		56–144		58–141					
	Mean	SD	Mean	SD	Mean	SD				
SSRT (ms)^c^	268.1	59.4	254.1	49	258.2	52.6	**6.012**	**.241**	**.046**	ADHD > Sibs = Controls
ICV (ms)^c^	.21	.051	.18	.047	.17	.042	**30.03**	**.555**	**<.001**	ADHD > Sibs > Controls
Errors (n)^c^	6.3	7.6	4.2	5.6	3.1	3.5	**13.56**	**.365**	**<.001**	ADHD > Sibs = Controls
Medication use (%)	77		0		0		**160.64**	**1.571**	**<.001**	ADHD > Sibs = Controls
Comorbid ODD d	55		4		0		**67.68**	**.876**	**<.001**	ADHD > Sibs = Controls
Comorbid CD d	12		0		0		**15.62**	**.393**	**<.001**	ADHD > Sibs = Controls
Comorbid RD d	34		11		11		**7.33**	**.267**	**.026**	ADHD > Controls

Note: ADHD = attention deficit/hyperactivity disorder; ODD = oppositional defiant disorder; CD = conduct disorder; RD = reading disability SSRT = stop-signal reaction time; ICV = intraindividual coefficient of variance; Errors = number of errors on go-trials. Bolded values indicate significant effects.

a ADHD diagnosis was based on K-SADS structured psychiatric interviews and Conners' questionnaires (Conners C.K. et al., 1998).

b Estimated IQ was based on the block-design and vocabulary subtests of the Wechsler Intelligence Scale for Children (WISC) or Wechsler Adult Intelligence Scale (WAIS-III) (Wechsler, 2002).

c Task effects for the stop-task derived from generalized estimate equation models, using a significance threshold of *p* < .05 and correcting for familiarity, gender age and IQ.

d ODD, CD and RD diagnosis was based on K-SADS structured psychiatric interviews (Kaufman et al., 1997).

**Table 2:** Region of interest coordinates.

Successful-stop network	*x*^a^	*y*	*z*	Wald-χ^2 b^	*p*-Value b	Between group difference
Left inferior frontal gyrus	−38	20	−18	16.34	<.001	Controls = Sibs > ADHD
Left superior frontal gyrus	−2	60	38	16.25	<.001	Controls = Sibs > ADHD
Failed-stop network:						
Left inferior frontal gyrus	−52	18	−12	35.29	<.001	Controls > Sibs > ADHD
Left superior frontal gyrus	−18	42	30	20.55	<.001	Controls > Sibs = ADHD

a Montreal Neurological Institute (MNI) space coordinates for peak voxels of the four regions of interest (ROIs).

b Wald-χ^2^, *p*-values and are derived from post-hoc generalized estimating equation models indicating the main diagnostic group effect on neural activation in these nodes.

**Table 3:** Connectivity patterns from the left inferior frontal gyrus seed region.

Left inferior frontal network	Valence	Side a	Peak voxel (MNI)			BA	*p*-Value b	# voxels c
*x*	*y*	*z*
Stop-success network								
Inferior frontal gyrus, pre-SMA and thalamus	+	R	48	14	−4	43–47, 13, 9, 6	<.0001	6037
Supramarginal area, fusiform gyrus	+	R	58	−36	28	40, 37	<.0001	4257
Fusiform gyrus	+	L	−40	−50	−28	37	<.0001	2959
Inferior frontal gyrus, insula and operculum	+	L	−48	8	−4	44, 13, 6	.0025	1752
Supramarginal area	+	L	−48	−38	34	40	.0386	1044
Medial prefrontal cortex	−	L/R	−10	64	24	38, 8–10	<.0001	6485
Precuneus	−	L/R	−10	−46	32	31	<.0001	4384
Lateral occipital lobe	−	L	−50	−64	28	39	.0049	1562
Superior temporal gyrus	−	L	−64	−34	−2	21, 22	.0280	1121
Stop-failed network								
Fusiform gyrus, cerebellum	+	L	−40	−48	−28	37, 19	<.0001	1957
Inferior frontal gyrus, insula	+	R	38	50	10	46, 47, 9	<.0001	1930
Temporal/parietal junction, fusiform gyrus	+	R	60	−36	24	40, 22, 21	.0002	1591
Dorsolateral prefrontal cortex	+	L	−50	42	22	46, 9	.0314	713
Temporal/parietal junction	+	L	−42	−30	20	41, 22, 13	.0378	686
Cerebellum	−	R	34	−82	−34	n.a.	.0096	891

Note: pre-SMA = pre-supplementary motor area; BA = Brodmann area.

a Side indicates the hemisphere (left/right).

b Correction for multiple comparisons applied using a cluster threshold of *z* > 2.3 and significance threshold of *p* < .05 corrected.

c # voxels indicates the number of voxels in a cluster.

**Table 4:** Connectivity patterns from the left superior frontal gyrus seed region.

Left SFG network	Valence	Side a	Peak voxel (MNI)			BA	*p*-Value b	# voxels c
*x*	*y*	*z*
Successful stop condition								
Lingual gyrus	+	L/R	−18	−72	6	19,18	<.0001	3178
Frontal pole, middle frontal gyrus	+	R	36	40	34	8–10, 46	.0094	877
Frontal pole, middle frontal gyrus	+	L	−38	44	28	8–10	.0132	827
Inferior frontal gyrus, insula, putamen	+	L	−40	0	20	47, 45, 13	.0574	616
Medial prefrontal cortex	−	L/R	−12	38	12	22,9,8,6	<.0001	4509
Precuneus	−	L/R	−6	−48	30	31	<.0001	2061
Lateral occipital cortex	−	L	−50	−60	26	40, 39, 22	<.0001	1629
Middle temporal gyrus	−	L	−62	−24	−8	21	.0002	1488
Lateral occipital cortex	−	R	52	−58	34	39	.0020	1122
Failed stop condition								
Inferior/medial frontal gyrus	+	R	46	32	20	45,46,9	.0002	1650
Middle temporal gyrus	+	R	56	−54	0	37	.0054	1062
Insula, caudate, anterior cingulate	+	L	−6	0	20	24,13	.0205	838
Temporal/parietal junction	+	R	54	−46	12	41,40	.0302	776
Frontal pole, superior frontal gyrus, anterior cingulate	−	R	24	44	18	32,24,9,8	<.0001	1754
Precuneus, lateral occipital cortex	−	L	−22	−52	20	41,40,31	.0002	1731
Frontal pole, superior frontal gyrus	−	L	−22	50	30	8–10	.0006	1471

Note: BA = Brodmann area.

a Side indicates the hemisphere (left/right).

b Correction for multiple comparisons applied using a cluster threshold of *z* > 2.3 and significance threshold of *p* < .05 corrected.

c # voxels indicates the number of voxels in a cluster.

**Table 5:** Group differences in connectivity patterns from the inferior frontal gyrus seed region.

Left inferior frontal network	Side a	Wald-chi^2 b^	Cohen's d	*p*-Value b	Peak voxel (MNI)			BA	# voxels c	Group averages d			Post-hoc comparisons e
*x*	*y*	*z*	ADHD	Siblings	Controls
Successful stop condition													
Cerebellum	L	16.498	0.405	<.001	−2	−74	−52	n. a.	589	.035 (.015)	−.061 (.019)	−.045 (.02)	Controls = Sibs < ADHD
Precuneus	L	29.313	0.549	<.001	−26	−74	0	18	238	.046 (.012)	−.048 (.013)	.039 (.018)	Sibs < ADHD = Controls
Anterior middle temporal gyrus	L	13.877	0.37	<.001	−68	−12	−20	21	216	−.015 (.012)	−.07 (.017)	.008 (.015)	Controls = Sibs < ADHD
Posterior middle temporal gyrus	L	12.861	0.356	.002	−70	−50	4	22	211	.035 (.014)	−.02 (.014)	−.05 (.013)	Controls < sibs < ADHD
Putamen	R	23.065	0.483	<.001	28	6	0	n. a.	162	−.017 (.012)	.071 (.014)	.052 (.015)	ADHD < Controls = Sibs
Failed stop condition													
Temporal pole	L	27.722	0.532	<.001	−28	−4	−36	21, 20	596	.028 (.011)	.017 (.016)	−.049 (.011)	Controls < Sibs = ADHD
Supramarginal gyrus	R	27.153	0.526	<.001	74	−20	24	40	266	.046 (.011)	.012 (.014)	−.0425 (.012)	Controls < Sibs = ADHD
Temporal pole	R	12.573	0.352	.002	44	−12	−30	20	207	.044 (.015)	.039 (.021)	−.0466 (.021)	Controls < Sibs = ADHD
Medial frontal gyrus, anterior cingulate	R	11.731	0.339	.003	14	50	2	32	196	.009 (.012)	−.031 (.014)	.048 (.019)	Sibs < ADHD = Controls
Cerebellum	L	10.15	0.315	.006	−8	−78	−48	n.a.	161	.02 (.021)	.042 (.032)	−.07 (.025)	Controls < Sibs = ADHD
Occipital cortex	L	15.001	0.385	<.001	−30	−82	36	19	157	−.028 (.016)	.067 (.019)	.036 (.02)	ADHD < Controls = Sibs
Inferior/middle frontal gyrus	R	20.806	0.457	<.001	36	18	32	9	155	−.003 (.012)	.066 (.012)	.001 (.014)	ADHD < Controls < Sibs
Superior frontal gyrus, preSMA	L/R	15.933	0.398	<.001	10	22	60	6	146	−.029 (.014)	−.037 (.018)	.06 (.02)	ADHD = Sibs < Controls
Middle temporal gyrus	L	18.142	0.425	<.001	−68	−56	0	37	145	−.024 (.01)	.041 (.014)	.034 (.013)	ADHD < Controls = Sibs

Note: BA = Brodmann area; pre-SMA = pre-supplementary motor area.

a Side indicates the hemisphere (left/right).

b Reported Wald-chi^2^ along with their *p*-values reflect the effect of diagnostic group on connectivity, derived from generalized estimating equation models corrected for familial dependency between siblings and covariate age, gender, IQ, and scan site. *p*-Values were adjusted for multiple comparisons using Bonferroni–Holm correction.

c # voxels indicates the number of voxels in a cluster.

d Means and standard errors for beta-values for the diagnostic groups.

e Post-hoc between-group differences in parameter estimates provided by generalized estimated equations model.

**Table 6:** Group differences in connectivity patterns from the superior frontal gyrus seed region.

Superior frontal network	Side a	Wald-chi^2 b^	Cohen's d	*p*-Value b	Peak voxel (MNI)			BA	# voxels c	Group averages d			Post-hoc comparisons e
*x*	*y*	*z*	ADHD	Siblings	Controls
Successful stop condition													
Precentral gyrus	L	21.296	0.463	<.001	−44	−4	54	6	620	.039 (.016)	−.054 (.012)	−.011 (.01)	Sibs < Controls < ADHD
Precentral gyrus	R	20.31	0.451	<.001	42	−8	60	6	572	.035 (.014)	−.046 (.011)	.013 (.011)	Sibs < ADHD = Controls
Frontal pole	R	20.196	0.45	<.001	36	48	−18	11	267	.054 (.015)	−.029 (.012)	−.036 (.016)	Controls = Sibs < ADHD
Thalamus	L	10.368	0.319	0.006	−16	−36	10	n. a.	173	−.044 (.016)	.018 (.01)	.027 (.011)	ADHD < Controls = Sibs
Anterior cingulate	R	16.754	0.408	<.001	6	32	14	24	155	.024 (.012)	−.055 (.014)	−.031 (.014)	Controls = Sibs < ADHD
Precuneus	L/R	17.59	0.419	<.001	12	−74	26	18, 7	136	.044 (.019)	−.045 (.012)	−.003 (.013)	Sibs < Controls < ADHD
Cerebellum	R	12.383	0.340	0.002	18	−62	−28	n. a.	121	.042 (.013)	−.017 (.007)	−.013 (.008)	Controls = Sibs < ADHD
Operculum	L	12.646	0.353	0.002	−26	−2	26	13	118	−.036 (.014)	.025 (.007)	.016 (.009)	ADHD < Controls = Sibs
Failed stop condition													
Inferior frontal gyrus	L	37.586	0.628	<.001	−40	44	18	9,10, 46	306	.033 (.021)	−.09 (.023)	.109 (.024)	Sibs = ADHD < Controls
Middle temporal gyrus	L	23.204	0.484	<.001	−50	2	−34	21	152	.031 (.026)	−.145 (.028)	−.108 (.029)	Controls = Sibs < ADHD

Note: BA = Brodmann area.

a Side indicates the hemisphere (left/right).

b Reported Wald-chi^2^ along with their *p*-values reflect the effect of diagnostic group on connectivity, derived from generalized estimating equation models corrected for familial dependency between siblings and covariate age, gender, IQ, and scan site. *p*-Values were adjusted for multiple comparisons using Bonferroni–Holm correction.

c # voxels indicates the number of voxels in a cluster.

d Means and standard deviations for beta-values for the diagnostic groups.

e Post-hoc between-group differences in parameter estimates provided by generalized estimated equations model.

## Appendix A Supplementary data

Altered neural connectivity patterns during response inhibition in adolescents with ADHD and their unaffected siblings: supplementary information.
