# Supplementary material for: Altered neural connectivity during response inhibition in adolescents with attention-deficit/hyperactivity disorder and their unaffected siblings
Source: Neuroimage Clin. 2015 Jan 13;7:325–35. doi: 10.1016/j.nicl.2015.01.004 (PMC4297885; doi:10.1016/j.nicl.2015.01.004)
Supplement: Supplementary file 1 — Altered neural connectivity patterns during response inhibition in adolescents with ADHD and their unaffected siblings: supplementary information. [file mmc1.docx]

**Altered neural connectivity patterns during response inhibition in adolescents with ADHD and their unaffected siblings: supplementary information**

**Supplementary Results**

Between group differences in fMRI activation

The selections of the regions of interest for the PPI analysis in this manuscript were based on the observed differences in neural activation between participants with ADHD, their unaffected siblings and healthy controls. The analyses providing these results, as well as their interpretation, are described in detail in (Van Rooij et al., 2014).

In short, three contrasts of interest were defined. The successful stop-go and failed stop-go contrasts were defined to isolate activation of successful and failed inhibition respectively, using go trial activity as an implicit baseline; and the third contrast, failed-successful stop, to model activation unique to the failed inhibition process.

The Between group differences in neural activation for the successful stop–go condition were located in the left inferior frontal, superior frontal and anterior cingulate gyrus, left supramarginal gyrus, right postcentral gyrus, and right temporal/parietal junction (SI Figure 1). For the failed stop–go condition, between group comparisons showed differences in left inferior and superior frontal, anterior cingulate, left supramarginal, and bilateral temporal/parietal area, as well as left cerebellum and right occipital areas (SI Figure 2). An overview of all overall group effects and differences between the three diagnostic groups is shown in SI Table 1. No effect was found for the successful – failed stop condition.


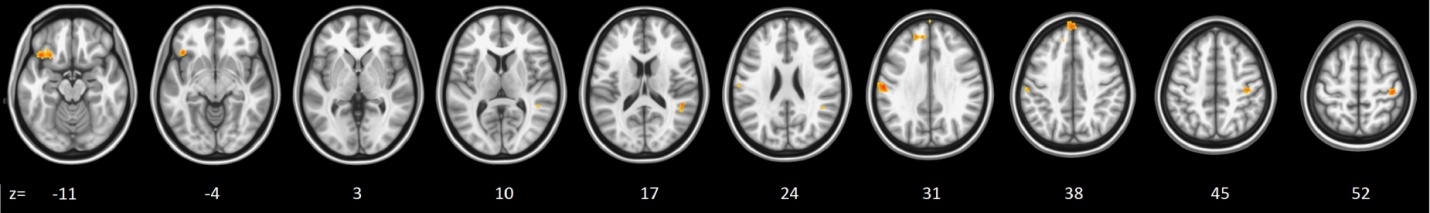


Supplementary Figure 1: Successful-stop network: Brain activation differences between controls and siblings or ADHD patients Red hues correspond to higher signal in control subjects. Right side of the image corresponds to the right hemisphere of the brain.


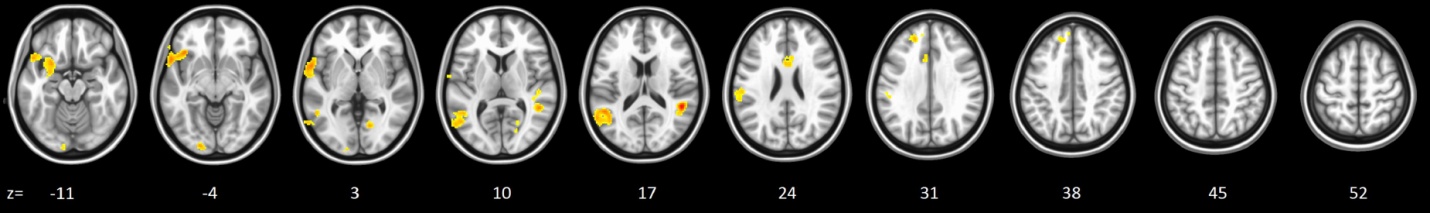


Supplementary Figure 2: Failed-stop network: Brain activation differences between controls and siblings or ADHD patients Red hues correspond to higher signal in control subjects. Right side of the image corresponds to the right hemisphere of the brain.

Supplementary Table 1. Brain areas with differential activation between diagnostic groups.

| Area ^b^ | side ^a^ | Mean *B* ^b^ | SD ^b^ | Wald-χ^2 c^ | p-value ^c^ | Cohen’s d ^c^ | peak voxel (MNI) | | | BA | # voxels ^d^ | between group effects ^c^ |  |
| --- | --- | --- | --- | --- | --- | --- | --- | --- | --- | --- | --- | --- | --- |
| Stop-success contrast | |  |  |  |  |  | x | y | z |  |  |  |  |
| Inferior frontal gyrus | L | 4.91 | 25.46 | 16.34 | <.001 | 0.402 | -38 | 20 | -18 | 44,47 | 371 | Controls = Sibs > ADHD |  |
| Superior frontal gyrus | L | -2.87 | 37.46 | 16.25 | <.001 | 0.401 | -2 | 60 | 38 | 8 | 245 | Controls = Sibs > ADHD |  |
| Supramarginal gyrus | L | -7.5 | 31.47 | 9.91 | 0.007 | 0.311 | -58 | -20 | 34 | 2,40 | 189 | Controls > Sibs = ADHD |  |
| Postcentral gyrus | R | -17.59 | 31.1 | 11.28 | 0.004 | 0.332 | 42 | -24 | 52 | 3,4 | 134 | Controls > Sibs = ADHD |  |
| Temporal-parietal junction | R | 17.22 | 21.34 | 7.1 | 0.029 | 0.262 | 48 | -42 | 14 | 41 | 95 | Controls > Sibs = ADHD |  |
| Stop-fail contrast | |  |  |  |  |  |  |  |  |  |  |  |  |
| Inferior frontal gyrus | L | 14.8 | 23.15 | 35.29 | <.001 | 0.606 | -52 | 18 | -12 | 13,44,47 | 1064 | Controls > Sibs > ADHD |  |
| Superior frontal gyrus | L | 3.34 | 24.51 | 20.55 | <.001 | 0.454 | -18 | 42 | 30 | 9 | 164 | Controls > Sibs = ADHD |  |
| Temporal-parietal junction | L | 14.97 | 25.02 | 22.46 | <.001 | 0.475 | -50 | -50 | -12 | 19,22 | 811 | Controls > Sibs > ADHD |  |
| Temporal-parietal junction | R | 11.81 | 16.12 | 33.5 | <.001 | 0.589 | 48 | -44 | 14 | 13 | 368 | Controls > Sibs > ADHD |  |
| Anterior cingulate cortex | L/R | 21.64 | 24.59 | 11.24 | 0.004 | 0.332 | -2 | 12 | 22 | 24 | 160 | Controls > Sibs = ADHD |  |
| Supramarginal gyrus | L | 16.44 | 30.61 | 10.57 | 0.005 | 0.321 | -58 | -24 | 26 | 40 | 151 | Controls = Sibs > ADHD |  |
| \| Note: BA = Brodmann area.  ^a^ Side indicates the hemisphere (left/right). ^b^ Activation clusters are derived from the F-contrasts testing differences in task activation as a function of diagnostic group, including gender,  IQ, age and scan-site as covariates. Correction for multiple comparisons performed using a cluster threshold of Z > 2.3 and a significance  threshold of p < .05 corrected  ^c^ Between group effects and associated Wald-χ^2^, p-values and cohen’s d reflect the specific diagnostic group in each region as derived from  post-hoc generalized estimating equation analyses, corrected for familial dependency between siblings as well as for covariates age, gender,  IQ, and scan site.  ^d^ # voxels indicates the number of voxels in a cluster. \| \| --- \| | | | | | | | | | | | | | |

In the successful-stop condition, siblings and probands showed less activation compared to controls in right temporal/parietal, left supramarginal, and right postcentral/supramarginal areas. In the left superior frontal and inferior frontal gyri, probands showed less activation compared to both siblings and controls, while the latter two did not differ.

In the failed-stop condition, we observed levels of activation for siblings that were in-between the levels observed for probands and controls, in bilateral temporal/parietal areas and the inferior frontal gyrus. In the anterior cingulate and left superior frontal gyri, probands and siblings showed similar levels of hypoactivation compared to controls. In the left supramarginal region, siblings did not differ from controls and showed higher activation compared to probands.

Group differences in connectivity patterns


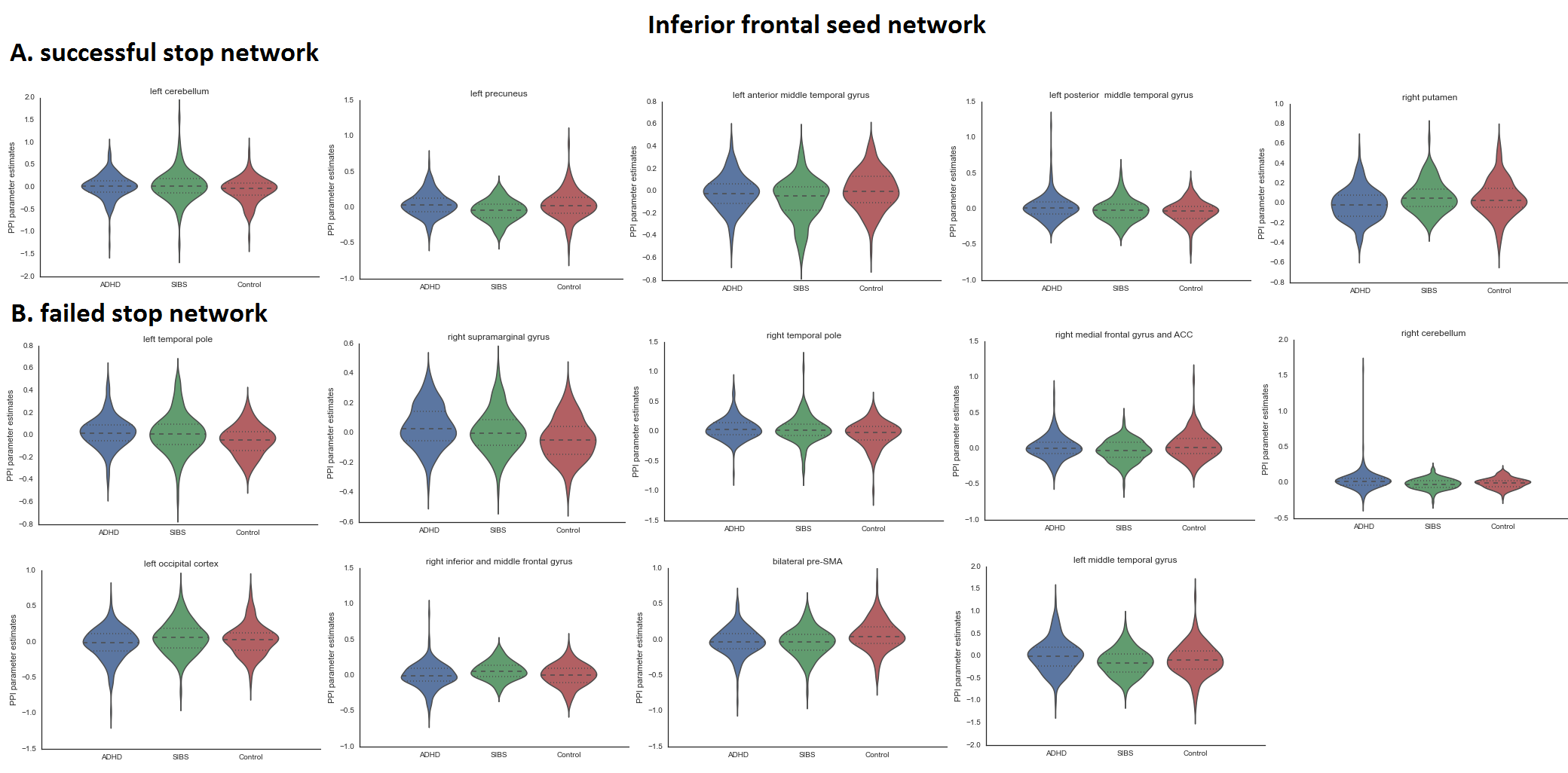
To further visualize the group differences in connectivity patterns from both seed regions, we have plotted the distribution of beta-values of all significant nodes from the main between-group contrast below (see Supplementary figures 3 and 4).

Supplementary Figure 3: Group differences in PPI connectivity betas per node for the inferior frontal seed region during the successful-stop contrast (A) and failed-stop contrast (B). Blue = ADHD, green = Siblings, red = controls.


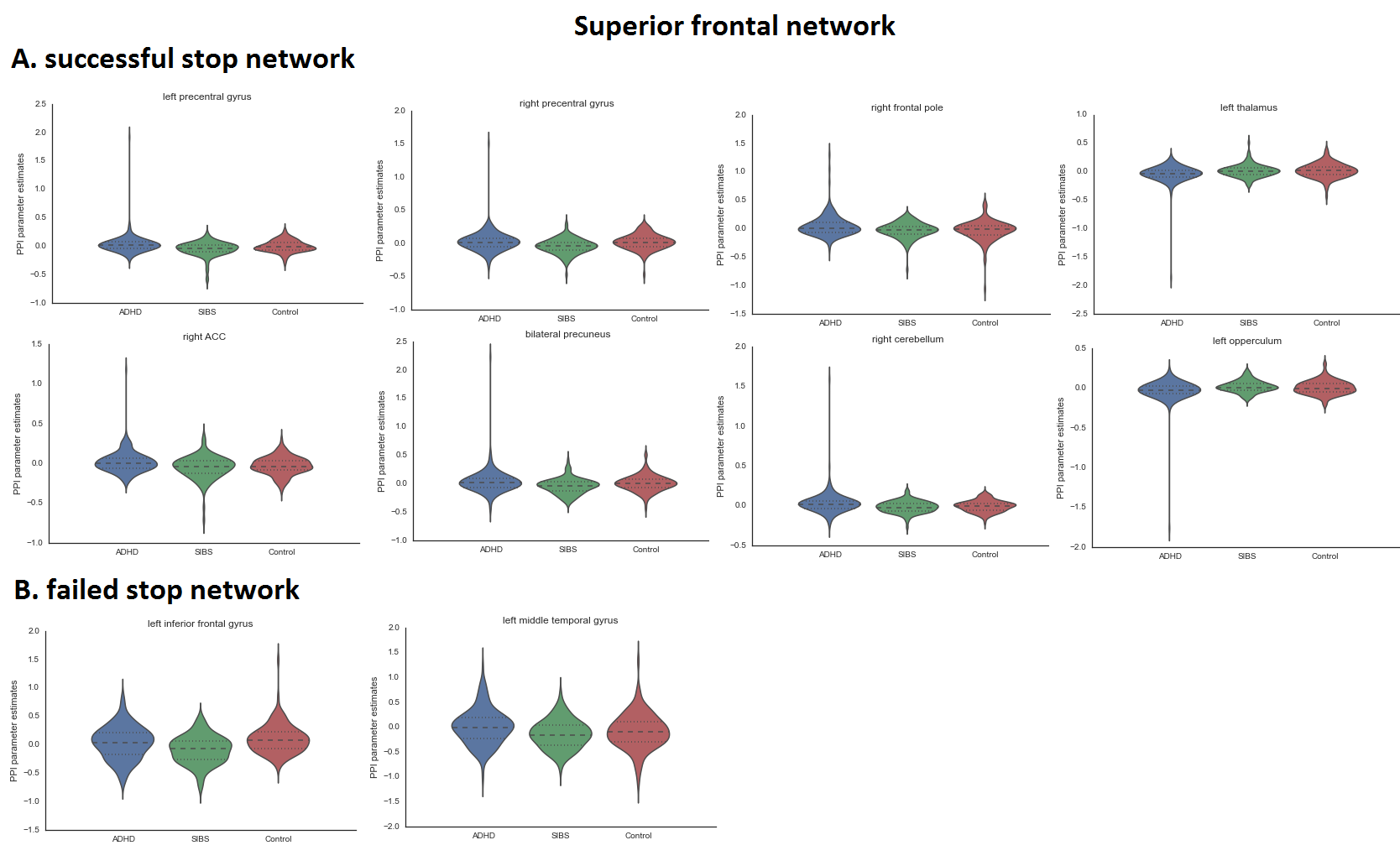
Supplementary Figure 4: Group differences in PPI connectivity betas per node for the superior frontal seed region during the successful-stop contrast (A) and failed-stop contrast (B). Blue = ADHD, green = Siblings, red = controls.

Influence of medication use and duration on PPI connectivity values

The main diagnostic group comparisons on the PPI beta-values from the main text of the paper were repeated included a continuous variable detailing the Cumulative Stimulant Intake (CSI) for each subject as a covariate. CSI is defined as the total lifetime intake of stimulant medication in mg, corrected for the treatment time. The results of this analysis (see Supplementary Table 2) indicate that none of the group effects on PPI connectivity were influenced by the inclusion of CSI in the model.

Additionally, to further examine the influence of medication use on PPI connectivity within participants with ADHD, an extra Generalized Estimating Equations models was run using CSI as a predicator and PPI beta values as dependent variable. This analysis showed a main effect of CSI on right connectivity between the left inferior frontal seed and right MFG/ ACC during failed stop-trials in participants with ADHD (χ^2^ =5.13, p=.023). However, this effect does not survive the correction for multiple comparisons. The results of this analysis indicate that the effect observed PPI connectivity values are largely unrelated to medication use.

Supplementary Table 2. Relations between medication use and PPI connectivity

| **Inferior frontal network** | Group effects on PPI connectivity, corrected for CSI | | | Association CSI and PPI connectivity in ADHD | |
| --- | --- | --- | --- | --- | --- |
| Stop-success network | side ^a^ | χ^2 b^ | p-value ^c^ | χ^2 b^ | p-value ^c^ |
| Cerebellum | L | 14.24 | <.001 | 0.30 | .583 |
| Precuneus | L | 30.24 | <.001 | 0.29 | .591 |
| anterior Middle Temporal Gyrus | L | 14.01 | <.001 | 0.16 | .211 |
| posterior Middle Temporal Gyrus | L | 18.52 | <.001 | 0.09 | .764 |
| Putamen | R | 20.01 | <.001 | 0.86 | .354 |
| Stop-failed network |  |  |  |  |  |
| Temporal Pole | L | 19.45 | <.001 | 0.72 | .398 |
| Supramarginal Gyrus | R | 26.41 | <.001 | 0.68 | .411 |
| Temporal Pole | R | 12.57 | .002 | 0.72 | .398 |
| MFG, ACC | R | 12.69 | .002 | 5.13 | .023 |
| Cerebellum | L | 10.27 | .006 | 0.03 | .861 |
| Occipital Cortex | R | 16.83 | <.001 | 0.40 | .528 |
| IFG, MFG | R | 18.01 | <.001 | 0.00 | .955 |
| SFG, preSMA | L/R | 16.08 | <.001 | 0.02 | .900 |
| Middle Temporal Gyrus | L | 21.87 | <.001 | 0.86 | .354 |
| **Superior frontal network** |  |  |  |  |  |
| Stop-success network | side ^a^ | χ^2 b^ | p-value ^c^ | χ^2 b^ | p-value ^c^ |
| Precentral Gyrus | L | 24.56 | <.001 | 1.94 | .164 |
| Precentral Gyrus | R | 23.53 | <.001 | 0.01 | .934 |
| Frontal Pole | R | 19.51 | <.001 | 0.01 | .913 |
| Thalamus | L | 10.76 | .005 | 0.24 | .624 |
| ACC | R | 16.22 | <.001 | 0.14 | .707 |
| Precuneus | L/R | 16.75 | <.001 | 2.28 | .131 |
| Cerebellum | R | 11.55 | .003 | 0.03 | .872 |
| Operculum | L | 12.86 | .002 | 1.41 | .235 |
| stop-failed network |  |  |  |  |  |
| IFG | L | 38.13 | <.001 | 0.91 | .340 |
| MTG | L | 22.45 | <.001 | 0.08 | .778 |
| Note: MTG = middle temporal gyrus; MFG = middle frontal gyrus. ACC = anterior cingulate gyrus; IFG = inferior frontal gyrus; preSMA = pre-supplementary motor area  ^a^ Side indicates the hemisphere (left/right).  ^b^ Reported statistics are derived from a single general estimated equations analysis corrected for familial dependency between siblings, and covariates age, gender, IQ and scan site.  ^c^ A Bonferroni-holm adjusted p-value was used to correct for multiple comparisons. | | | | | |

Associations of stop-signal task outcomes and ADHD severity and FD with PPI connectivity values

Additional GEE analyses were performed on the exported PPI beta values from the main group contrast, in order to investigate the association between PPI connectivity and Stop-task performance (See Supplementary Table 3), as well as between PPI connectivity and ADHD severity (see Supplementary Table 4) and PPI connectivity and the frame-wise displacement (FD) of subjects over all scans (see Supplementary Table 5).

The results of these analyses indicate that connectivity between the inferior frontal seed and posterior medial temporal gyrus is significantly associated with both ICV (*B*=.792, χ^2^=15.827, p<.001) and SSRT (*B*=.011, χ^2^=6.881, p=.009). Connectivity between the superior frontal seed and left thalamus is also associated with the Error rates (*B*=.05, χ^2^=7.978, p=.005). Furthermore, PPI connectivity values are associated with ADHD severity as measured by the Conners’ questionnaire scores in almost all nodes showing a main group effect, and the connectivity between the left inferior frontal seed and posterior medial temporal gyrus and right inferior frontal gyrus is correlated with the FD over all scans.

Supplementary Table 3. Relations between connectivity values and task outcome measures from the Inferior Frontal seed region network

| **Inferior frontal network** | |  | ICV | | |  | SSRT | | |  | Errors | | |
| --- | --- | --- | --- | --- | --- | --- | --- | --- | --- | --- | --- | --- | --- |
| Stop-success condition | side ^a^ | R^2 b^ | *B* ^b^ | χ^2 b^ | p-value ^c^ | R^2 b^ | *B* | χ^2^ | p-value | R^2 b^ | *B* | χ^2^ | p-value |
| Cerebellum | L | .001 | .377 | 2.854 | .0910 | .001 | .0001 | .606 | .436 | .001 | -.002 | .670 | .413 |
| Precuneus | L | .001 | .600 | .114 | .7360 | .001 | -.0001 | .658 | .417 | .002 | .002 | 1.005 | .316 |
| Anterior MTG | L | .001 | .133 | .531 | .4660 | .001 | .0001 | .124 | .725 | .001 | .0003 | .042 | .838 |
| Posterior MTG | L | **.044** | **.792** | **15.827** | **<.001** | **.011** | **.0003** | **6.881** | **.009** | .003 | -.002 | 2.807 | .094 |
| Putamen | R | .004 | -.172 | 1.003 | .3170 | .003 | -.001 | .200 | .654 | .007 | -.001 | .625 | .429 |
| Stop-failed condition |  |  |  |  |  |  |  |  |  |  |  |  |  |
| Temporal pole | L | .002 | .205 | 1.720 | .1900 | .007 | .0001 | 1.146 | .284 | .004 | .001 | .560 | .454 |
| Supramarginal gyrus | R | .001 | .047 | .106 | .7440 | .005 | .0001 | 1.303 | .254 | .003 | .0004 | .071 | .789 |
| Temporal pole | R | .005 | .233 | 1.488 | .2220 | .001 | .0001 | .005 | .943 | .004 | .001 | 1.488 | .222 |
| MFG, ACC | R | .004 | -.232 | 2.004 | .1570 | .001 | -.0001 | .465 | .495 | .006 | .003 | 5.513 | .019 |
| Cerebellum | L | .002 | .319 | 1.102 | .3130 | .007 | .0002 | 1.569 | .210 | .001 | -.002 | .276 | .599 |
| Occipital cortex | R | .001 | -.034 | .200 | .8860 | .001 | .0001 | .358 | .550 | .001 | .0001 | .048 | .826 |
| IFG, MFG | R | .002 | .110 | .376 | .5400 | .003 | -.0001 | 1.202 | .273 | .001 | -.0002 | .021 | .885 |
| SFG, preSMA | L/R | .003 | -.252 | 1.521 | .2170 | .001 | -.0001 | .061 | .805 | .001 | .0001 | .002 | .964 |
| Middle temporal gyrus | L | .001 | -.024 | .240 | .8780 | .001 | .0001 | .178 | .673 | .001 | .0001 | .015 | .903 |
| **Superior frontal network** | |  |  | | |  | | | |  | | | |
| Stop-success condition | side ^a^ | R^2^ | *B* | χ2 | p-value | R^2^ | *B* | χ2 | p-value | R^2^ | *B* | χ2 | p-value |
| Precentral gyrus | L | .001 | .074 | .240 | .624 | .002 | .0001 | .253 | .615 | .001 | .0003 | .074 | .785 |
| Precentral gyrus | R | .001 | -.120 | .006 | .936 | .001 | -.0001 | .584 | .445 | .001 | .001 | .479 | .489 |
| Frontal pole | R | .002 | .270 | 1.885 | .170 | .001 | -.00001 | .005 | .943 | .008 | .005 | 2.336 | .126 |
| Thalamus | L | .001 | -.097 | .425 | .514 | .001 | -.0001 | .587 | .444 | **.05** | **-.002** | **7.978** | **.005** |
| ACC | R | .001 | .810 | .331 | .564 | .003 | .0001 | .227 | .634 | .002 | .001 | .713 | .398 |
| Precuneus | L/R | .001 | -.130 | .784 | .376 | .003 | .00001 | .769 | .381 | .002 | .001 | .784 | .376 |
| Cerebellum | R | .001 | .014 | .013 | .909 | .001 | -.0001 | .454 | .500 | .001 | -.0001 | .068 | .794 |
| Operculum | L | .001 | .029 | .055 | .814 | .007 | -.00001 | 4.710 | .030 | .002 | -.001 | 3.740 | .053 |
| Stop-failed condition |  |  |  |  |  |  |  |  |  |  |  |  |  |
| IFG | L | .002 | -.440 | 2.043 | .153 | .002 | -.0003 | 1.909 | .167 | .001 | .004 | 2.928 | .087 |
| MTG | L | .001 | .137 | .149 | .700 | .001 | .0001 | .097 | .755 | .001 | .001 | .071 | .790 |
| Note: SSRT = stop-signal reaction time; ICV = Intraindividual Coefficient of Variance; Errors = number of errors on go-trials; MTG = middle temporal gyrus; MFG = middle frontal gyrus. ACC = anterior cingulate gyrus; IFG = inferior frontal gyrus. Bolded values indicate significant effects.  ^a^ Side indicates the hemisphere (left/right).  ^b^ Reported statistics are derived from a single general estimated equations analysis corrected for familial dependency between siblings, and covariates age, gender, IQ and scan site.  ^c^ A Bonferroni-holm adjusted p-value was used to correct for multiple comparisons. | | | | | | | | | | | | | |


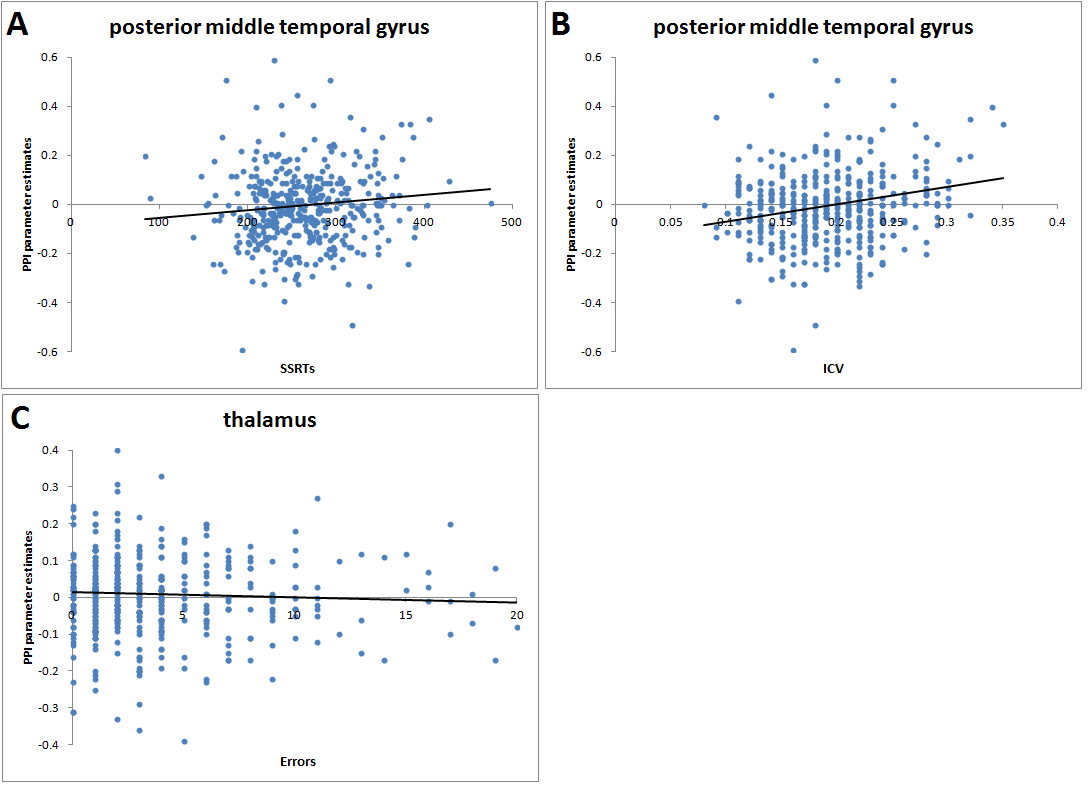
Supplementary Figure 5: PPI connectivity betas values between the left inferior frontal seed node and the left posterior middle temporal gyrus are associated with SSRT (A) and ICV (B) values. PPI connectivity values between the left superior frontal seed node and the left thalamus are associated with Error scores (C).

Supplementary Table 4. Relations between connectivity values and symptom scores

| **Inferior frontal network** | | Conners Scores ^b^ | | |
| --- | --- | --- | --- | --- |
| Stop-success condition | side ^a^ | *B* | χ2 | p-value ^c^ |
| Cerebellum | L | **.038** | **11.914** | **.001** |
| Precuneus | L | **.025** | **5.933** | **.015** |
| Anterior MTG | L | .011 | .952 | .329 |
| Posterior MTG | L | **.260** | **7.289** | **.007** |
| Putamen | R | -.017 | 4.584 | .058 |
| Stop-failed condition |  |  |  |  |
| Temporal pole | L | **.027** | **9.502** | **.002** |
| Supramarginal gyrus | R | **.030** | **14.943** | **<.001** |
| Temporal pole | R | .023 | 2.912 | .088 |
| MFG, ACC | R | -.011 | 1.374 | .241 |
| Cerebellum | L | **.035** | **4.688** | **.030** |
| Occipital cortex | R | -.019 | 2.439 | .118 |
| IFG, MFG | R | -.002 | .058 | .810 |
| SFG, preSMA | L/R | **-.024** | **5.049** | **.025** |
| Middle temporal gyrus | L | **-.021** | **5.824** | **.016** |
| **Superior frontal network** | |  |  |  |
| Stop-success condition | side ^a^ | *B* | χ2 | p-value ^c^ |
| Precentral gyrus | L | **.025** | **8.497** | **.004** |
| Precentral gyrus | R | **.027** | **11.298** | **.001** |
| Frontal pole | R | **.038** | **18.561** | **<.001** |
| Thalamus | L | **-.027** | **9.482** | **.002** |
| ACC | R | **.024** | **9.474** | **.002** |
| Precuneus | L/R | **.024** | **6.349** | **.012** |
| Cerebellum | R | **.020** | **8.633** | **.003** |
| Operculum | L | **-.020** | **8.318** | **.004** |
| Stop-failed contition |  |  |  |  |
| IFG | L | -.023 | 1.776 | .183 |
| MTG | L | **.054** | **8.146** | **.004** |
| Note: ACC= anterior cingulate gyrus; MTG = middle temporal gyrus; MFG = middle frontal gyrus. ACC = anterior cingulate gyrus; IFG = inferior frontal gyrus. Bolded values indicate significant effects.  ^a^ Side indicates the hemisphere (left/right).  ^b^ Combined scores on the Conner’s Parent Rating Scales (CPRS) and Conners’ Adult ADHD Rating Scale (CAARS) or Conners’ Teacher Rating Scales (CTRS).  ^c^ Reported statistics are derived from a single general estimated equations analysis corrected for familial dependency between siblings, and covariates age, gender, IQ and scan site. | | | | |


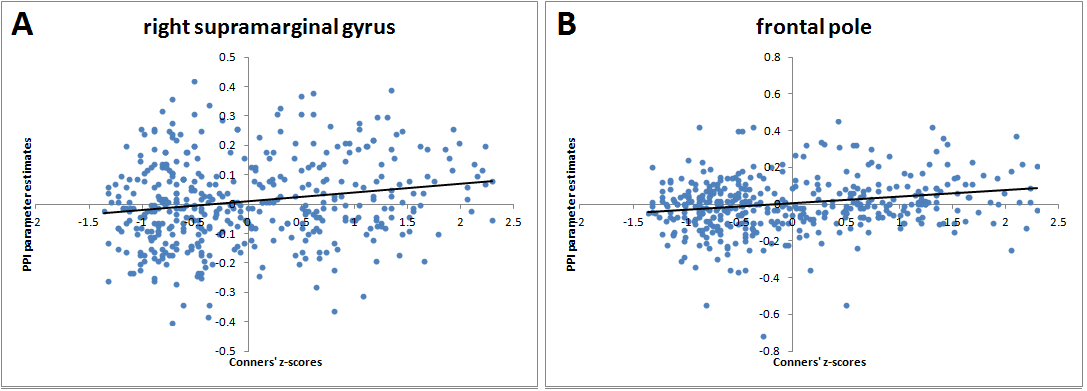
Supplementary Figure 6: PPI connectivity betas values between the left inferior frontal seed node and the right supramarginal gyrus (A) as well as between the left superior frontal seed node and the right frontal pole (B) are associated Conners scores.

Supplementary Table 5. Relations between connectivity values and the root-mean-square of the total frame wise displacement across all subjects

| **Left inferior frontal network** |  |  |  |  |
| --- | --- | --- | --- | --- |
| Stop-success condition | side ^a^ | B ^b^ | Wald-chi^2 b^ | p-value ^b^ |
| Cerebellum | L | .115 | 1.05 | .306 |
| Precuneus | L | -.114 | 3.24 | .072 |
| Anterior MTG | L | .007 | 0.01 | .905 |
| Posterior MTG | L | **.167** | **8.42** | **.004** |
| Putamen | R | .080 | 1.31 | .253 |
| Stop-failed condition |  |  |  |  |
| Temporal pole | L | .044 | 0.34 | .563 |
| Supramarginal gyrus | R | .009 | 0.04 | .837 |
| Temporal pole | R | .189 | 3.41 | .065 |
| MFG, ACC | R | .030 | 0.15 | .703 |
| Cerebellum | L | .153 | 0.63 | .428 |
| Occipital cortex | R | .033 | 0.16 | .693 |
| IFG, MFG | R | **.112** | **4.86** | **.027** |
| SFG, preSMA | L/R | -.080 | 1.16 | .281 |
| Middle temporal gyrus | L | -.008 | 0.02 | .880 |
| **Superior frontal network** |  |  |  |  |
| Stop-success network | side | B | Wald-chi^2^ | p-value |
| Precentral gyrus | L | -.045 | 0.04 | .550 |
| Precentral gyrus | R | -.059 | 0.97 | .324 |
| Frontal pole | R | .075 | 1.04 | .308 |
| Thalamus | L | -.026 | 0.35 | .553 |
| ACC | R | .040 | 1.03 | .311 |
| Precuneus | L/R | -.056 | 0.78 | .376 |
| Cerebellum | R | -.023 | 0.42 | .515 |
| Operculum | L | .024 | 0.39 | .532 |
| stop-failed network |  |  |  |  |
| IFG | L | .113 | 1.18 | .277 |
| MTG | L | -.061 | 0.23 | .630 |
| Note: MTG = middle temporal gyrus; MFG = middle frontal gyrus. ACC = anterior cingulate gyrus; IFG = inferior frontal gyrus. Bolded values indicate significant effects, before Bonferroni-Holm correction.  ^a^ Side indicates the hemisphere (left/right).  ^b^ Reported statistics are derived from a single general estimated equations analysis corrected for familial dependency between siblings, and covariates age, gender, IQ and scan site. | | | | |

**References:**

Van Rooij, D., Hartman, C. A., Mennes, M., Oosterlaan, J., Franke, B., Rommelse, N., Heslenfeld, D., et al. (2014). Neural activation patterns in inferior frontal areas during response inhibition distinguish adolescents with ADHD, their unaffected siblings, and healthy controls. *American Journal of Psychiatry*, *in press*.
